# Mr. William Howells Rix

**Published:** 1910-06-04

**Authors:** 


We have to record the death of Mr. William Howells Rix, in his seventy-fourth year. Mr. Rix studied at the Middlesex Hospital and became a M.R.C.S.Eng. in 1858, and an L.S.A. in 1859. At Middlesex Hospital he was at one time house surgeon, and his other appointments include that of consulting surgeon to the Tunbridge Wells General Hospital.

